# Interdependent YpsA- and YfhS-Mediated Cell Division and Cell Size Phenotypes in *Bacillus subtilis*

**DOI:** 10.1128/mSphere.00655-20

**Published:** 2020-07-22

**Authors:** Robert S. Brzozowski, Brooke R. Tomlinson, Michael D. Sacco, Judy J. Chen, Anika N. Ali, Yu Chen, Lindsey N. Shaw, Prahathees J. Eswara

**Affiliations:** a Department of Cell Biology, Microbiology, and Molecular Biology, University of South Florida, Tampa, Florida, USA; b Department of Molecular Medicine, University of South Florida, Tampa, Florida, USA; University of Iowa

**Keywords:** FtsZ, GpsB, filamentation, SLOG, cell shape, cell morphology, MreB, PBP, peptidoglycan

## Abstract

Although many bacterial cell division factors have been uncovered over the years, evidence from recent studies points to the existence of yet-to-be-discovered factors involved in cell division regulation. Thus, it is important to identify factors and conditions that regulate cell division to obtain a better understanding of this fundamental biological process. We recently reported that in the Gram-positive organisms Bacillus subtilis and Staphylococcus aureus, increased production of YpsA resulted in cell division inhibition. In this study, we isolated spontaneous suppressor mutations to uncover critical residues of YpsA and the pathways through which YpsA may exert its function. Using this technique, we were able to isolate four unique intragenic suppressor mutations in *ypsA* (E55D, P79L, R111P, and G132E) that rendered the mutated YpsA nontoxic upon overproduction. We also isolated an extragenic suppressor mutation in *yfhS*, a gene that encodes a protein of unknown function. Subsequent analysis confirmed that cells lacking *yfhS* were unable to undergo filamentation in response to YpsA overproduction. We also serendipitously discovered that YfhS may play a role in cell size regulation. Finally, we provide evidence showing a mechanistic link between YpsA and YfhS.

**IMPORTANCE** Bacillus subtilis is a rod-shaped Gram-positive model organism. The factors fundamental to the maintenance of cell shape and cell division are of major interest. We show that increased expression of *ypsA* results in cell division inhibition and impairment of colony formation on solid medium. Colonies that do arise possess compensatory suppressor mutations. We have isolated multiple intragenic (within *ypsA*) mutants and an extragenic suppressor mutant. Further analysis of the extragenic suppressor mutation led to a protein of unknown function, YfhS, which appears to play a role in regulating cell size. In addition to confirming that the cell division phenotype associated with YpsA is disrupted in a *yfhS*-null strain, we also discovered that the cell size phenotype of the *yfhS* knockout mutant is abolished in a strain that also lacks *ypsA*. This highlights a potential mechanistic link between these two proteins; however, the underlying molecular mechanism remains to be elucidated.

## Abstract (precis)

Bacillus subtilis is a rod-shaped Gram-positive model organism. The factors fundamental to the maintenance of cell shape and cell division are of major interest. We show that increased expression of *ypsA* results in cell division inhibition and impairment of colony formation on solid medium. Colonies that do arise possess compensatory suppressor mutations. We have isolated multiple intragenic (within *ypsA*) mutants and an extragenic suppressor mutant. Further analysis of the extragenic suppressor mutation led to a protein of unknown function, YfhS, which appears to play a role in regulating cell size. In addition to confirming that the cell division phenotype associated with YpsA is disrupted in a *yfhS*-null strain, we also discovered that the cell size phenotype of the *yfhS* knockout mutant is abolished in a strain that also lacks *ypsA*. This highlights a potential mechanistic link between these two proteins; however, the underlying molecular mechanism remains to be elucidated.

## INTRODUCTION

Bacterial cell division is an essential process orchestrated by a multitude of cell division proteins (1). During growth an essential cell division protein FtsZ, forms a ring-like structure and marks the site of division. There, it serves in the recruitment of additional divisome proteins and commences septation (2, 3). Although known FtsZ regulatory systems, such as the Min system and nucleoid occlusion, have been well characterized (4), recent studies have determined that correct cell division site selection can occur independent of these mechanisms in both Bacillus subtilis and Escherichia coli (5, 6). These findings highlight the need to investigate and discover other factors involved in regulating cell division in bacteria. In our lab, we have identified a potential cell division regulator in B. subtilis and Staphylococcus aureus, YpsA (7).

YpsA is conserved in the *Firmicutes* phylum of Gram-positive bacteria and appears to be in a syntenous relationship with a known cell division protein, GpsB (7). The crystal structure of B. subtilis YpsA was solved by a structural genomics group in 2006 (PDB ID 2NX2) (8). Based on the structural features, YpsA was placed as the founding member of the “YpsA proper” subclade within the SLOG (SMF/DprA/*LOG*) protein superfamily (9), and yet the precise function of YpsA remains to be elucidated. The structure of YpsA resembles that of DprA (the root mean square deviation with DprA of Helicobacter pylori is 2.79 Å [PDB ID 4LJR]), another member of the SLOG superfamily, which is a single-stranded DNA-binding protein involved in DNA recombination (10, 11). Previously, we found that YpsA provides oxidative stress protection in B. subtilis and that overproduction of YpsA results in cell division inhibition, through FtsZ mislocalization, in a growth rate-dependent manner (7). We showed that the YpsA-GFP fusion is functional and forms intracellular foci. Focus formation appears to be a prerequisite for filamentation; however, its physiological significance remains unknown. In addition, using site-directed mutagenesis, we identified multiple amino acid residues that are potentially important for the structure and/or function of YpsA, including residues located in the conserved substrate binding pocket made up of glycine and glutamate residues predicted by Burroughs et al. (9). In addition, we have shown that the potential function of YpsA in cell division is also conserved in the Gram-positive pathogen S. aureus (7).

In this study, we utilized a classic spontaneous suppressor isolation technique for further identification of critical amino acid residues that are important for YpsA structure and/or function, as well as for the elucidation of the molecular mechanism through which YpsA acts. By screening for suppressor mutations of a lethal YpsA overproduction phenotype, we were able to isolate and characterize four unique intragenic suppressor mutations (E55D, P79L, R111P, and G132E). Each of these mutations was found to prevent lethality and related cell division inhibition. Focus formation was also impaired in the P79L, R111P, and G132E mutants. In addition, we also identified an extragenic suppressor mutation that introduced a premature stop codon in the *yfhS* gene, which codes for a protein of unknown function. Upon subsequent analysis, we verified that YpsA-dependent cell division inhibition is abolished in cells lacking *yfhS*. Here, we speculate the possible ways by which YfhS- and YpsA-mediated cell division phenotypes could be linked. Interestingly, during the course of our experiments, we discovered that YfhS may play a role in cell size regulation, since *yfhS*-null cells are significantly smaller in cell width and length compared to the wild-type control.

## RESULTS

### Overexpression of *ypsA* results in a growth defect on solid medium.

**FIG 1:**
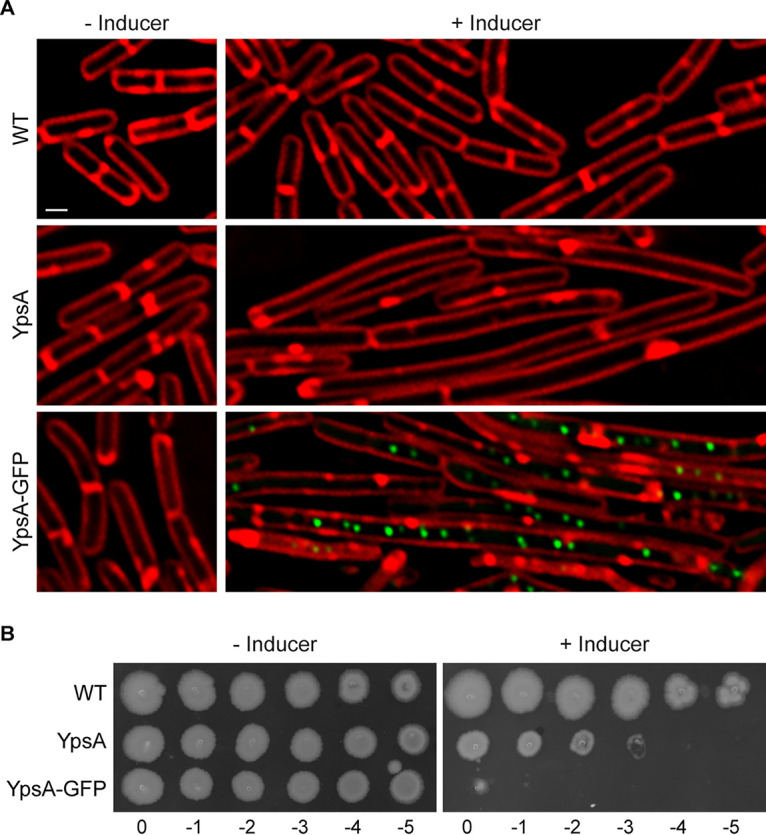
Overproduction of YpsA or YpsA-GFP results in a lethal phenotype. (A) Cell morphology of wild-type cells (PY79; WT) and cells harboring an IPTG-inducible copy of either *ypsA* (GG82) or *ypsA-gfp* (GG83) grown in the absence of inducer or in the presence of 250 μM IPTG for 1 h. The fluorescence levels of red membrane stain (FM4-64; red) and GFP (green) are shown. Scale bar, 1 μm. (B) Spot assays of WT cells (PY79) and cells containing an inducible copy of either *ypsA* (GG82) or *ypsA-gfp* (GG83). Serially diluted standardized cultures were spotted on plates containing no inducer (left panel) or 1 mM IPTG (right panel) and grown overnight at 37°C. Corresponding dilution factors are indicated below.

We have previously shown that overproduction of either YpsA or YpsA-GFP results in severe filamentation in B. subtilis (7) (Fig. 1A), a phenotype that is characteristic of cell division inhibition in this organism. Consistent with our earlier report, we noticed that YpsA-GFP forms distinct intracellular foci upon overproduction (Fig. 1A; see YpsA-GFP panel). Further analysis with DNA labeling revealed that these foci appear to be nucleoid-associated (see Fig. S1A in the supplemental material). To test whether filamentous growth in the presence of inducer results in a distinguishable phenotype on solid medium, we conducted a spot assay. Briefly, serial dilutions of exponentially growing wild-type (WT) cells, and cells containing an IPTG (isopropyl-β-d-thiogalactopyranoside)-inducible copy of either *ypsA* or *ypsA-gfp* were spotted on solid growth medium with or without inducer. In the absence of inducer, all strains of all tested dilutions grew similar to the WT control (Fig. 1B, see left panel). In the presence of inducer, we observed a significant growth defect associated with both YpsA and YpsA-GFP overproduction, suggesting that cell division inhibition caused by *ypsA* or *ypsA-gfp* overexpression was lethal (Fig. 1B, see right panel). Interestingly, YpsA-GFP overproduction resulted in a more severe growth phenotype compared to untagged YpsA. To test whether this difference is due to increased accumulation of YpsA-GFP in the cells, we tagged both YpsA and YpsA-GFP with an FLAG tag at their C termini and conducted an anti-FLAG Western blot analysis. Overproduction of YpsA-FLAG and YpsA-GFP-FLAG resulted in filamentation and a growth defect on solid medium that was of similar extent compared to their non-FLAG-tagged counterparts (Fig. S1B and C). The ratios of YpsA-GFP-FLAG and SigA (internal loading control) were similar to those of YpsA-FLAG and SigA (0.68 and 0.80, respectively; Fig. S1D), suggesting that the increased lethality of the green fluorescent protein (GFP)-tagged version is not due to any changes in accumulation. Next, we utilized the severe lethality elicited by YpsA-GFP overproduction as a tool to isolate spontaneous suppressors.

10.1128/mSphere.00655-20.1 FIG S1 Analysis of YpsA localization and accumulation. (A) Micrographs of WT (PY79) and YpsA-GFP (GG83; grown in the presence or absence of 250 μM IPTG for 1 h) cells showing fluorescence of FM4-64 (membrane), DAPI (DNA), GFP, and overlay. (B) Fluorescence micrographs of strains containing an IPTG-inducible copy of either *ypsA-flag* (RB121) or *ypsA-gfp-flag* (RB125) grown in the absence of inducer (left panels) or in the presence of 250 μM IPTG for 1 h (right panels). Fluorescence of FM4-64 membrane dye (red) and GFP (green) are shown. Scale bar, 1 μm. (C) Spot assays including wild-type cells (PY79) and cells containing an IPTG-inducible copy of either *ypsA* (GG82), *ypsA-gfp* (GG83), *ypsA-flag* (RB121), or *ypsA-gfp-flag* (RB125). Dilutions of standardized cultures were spotted on solid medium without inducer (top panel) or containing 1 mM IPTG (bottom panel). Corresponding dilution factors are indicated below. (D) Anti-FLAG and anti-SigA (loading control) immunoblots of RB121 (YpsA-FLAG), RB412 (Δ*yfhS* + YpsA-FLAG), RB125 (YpsA-GFP-FLAG), and RB413 (Δ*yfhS* + YpsA-GFP-FLAG) cell lysates. The FLAG/SigA ratios corresponding to each lane are shown. Download FIG S1, PDF file, 1.2 MB. Copyright © 2020 Brzozowski et al. 2020 Brzozowski et al. This content is distributed under the terms of the Creative Commons Attribution 4.0 International license.

### Isolation of spontaneous suppressor mutations.

**FIG 2:**
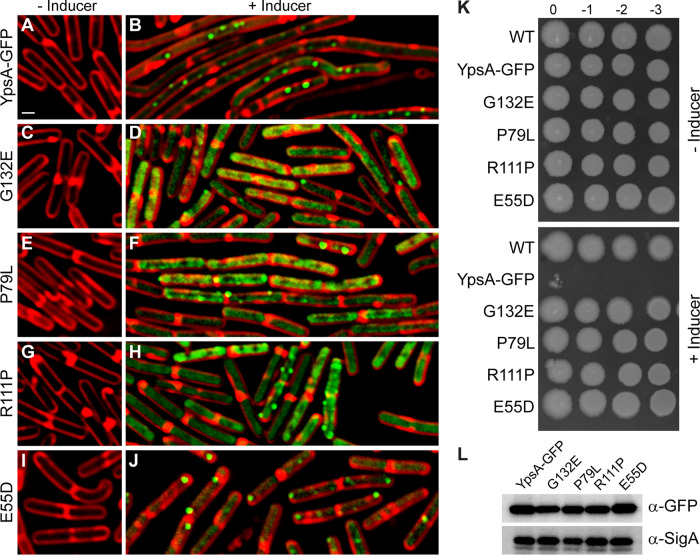
Isolation of spontaneous suppressors. (A to J) Fluorescence microscopy comparing cells containing an inducible copy *ypsA*-*gfp* (GG83) and cells containing intragenic mutations (ypsA*-gfp) isolated during the suppressor screen that resulted in single amino acid changes: G132E (RB300), P79L (RB301), R111P (RB328), and E55D (RB327). Cells were grown in the absence (A, C, E, G, and I) or presence (B, D, F, H, and J) of 250 μM IPTG for 1 h. Fluorescence of FM4-64 (red) and GFP (green) is shown. Scale bar, 1 μm. (K) Spot assays of strains harboring an IPTG-inducible copy of *ypsA-gfp* (GG83) or ypsA*-gfp (RB300, RB301, RB328, and RB327) grown without inducer (top panel) or with 1 mM IPTG (bottom panel). Corresponding dilution factors are shown on top. (L) The stability of YpsA-GFP and YpsA*-GFP variants was confirmed when cells were grown in the presence of inducer. Cell lysates were probed via immunoblotting using anti-GFP and anti-SigA (loading control) antisera.

The YpsA-GFP-overproducing strain was streaked out for single colony isolation on multiple inducer containing plates, and the plates were incubated overnight as described in Materials and Methods. Only a few colonies formed per plate, presumably due to spontaneous suppressor mutations, which allow for normal growth despite the presence of inducer. After multiple iterations of suppressor isolation, likely mutations were subsequently determined to be either intragenic (within inducible *ypsA-gfp*, henceforth noted as ypsA*-gfp for simplicity) or extragenic (elsewhere on the chromosome), and their chromosomes were sequenced to identify the mutations (see Fig. S2 in the supplemental material). Using this approach, we were able to isolate four unique intragenic suppressor mutations: G132E, P79L, R111P, and E55D (listed in the order of isolation). Immunoblotting indicated that these mutant versions of YpsA were stably produced (Fig. 2L). Next, fluorescence microscopy was used to determine whether these mutations were able to rescue the lethal filamentous phenotype observed when unmutated YpsA-GFP was overproduced. This was carried out on exponentially growing cells of the *ypsA-gfp* overexpression strain and all ypsA*-gfp intragenic suppressor strains in the absence or presence of inducer. In the absence of inducer, all strains exhibited similar cell lengths (YpsA-GFP, 3.23 ± 0.74 μm [Fig. 2A]; G132E, 3.49 ± 0.93 μm [Fig. 2C]; P79L, 3.24 ± 0.81 μm [Fig. 2E]; R111P, 3.16 ± 0.76 μm [Fig. 2G]; E55D, 3.23 ± 0.83 μm [Fig. 2I]; *n* = 100 for all cell length measurements). Upon the addition of inducer, we found that cells overexpressing ypsA*-gfp did not exhibit filamentation, unlike the *ypsA-gfp* control (YpsA-GFP, 8.93 ± 5.67 μm [Fig. 2B]; G132E, 2.99 ± 0.88 μm [Fig. 2D]; P79L, 3.29 ± 0.81 μm [Fig. 2F]; R111P, 3.06 ± 0.98 μm [Fig. 2H]; E55D, 3.10 ± 0.83 μm [Fig. 2J]), indicating that the intragenic suppressors were unable to elicit filamentation upon overproduction. We also noted that G132E, P79L, and R111P suppressors displayed impaired focus formation in comparison to the *ypsA-gfp* control (Fig. 2B, D, F, and H). However, focus formation in the E55D suppressor was not impaired (Fig. 2J).

10.1128/mSphere.00655-20.2 FIG S2 Flow chart detailing the methodology used to screen spontaneous suppressor mutations. Download FIG S2, PDF file, 0.7 MB. Copyright © 2020 Brzozowski et al. 2020 Brzozowski et al. This content is distributed under the terms of the Creative Commons Attribution 4.0 International license.

Each of the intragenic suppressors were subjected to a spot assay to test whether these point mutations were able to grow normally in contrast to the *ypsA-gfp* overexpression strain that displayed a lethal phenotype on solid medium in the presence of inducer. In the absence of inducer, all strains grew similar to the WT control (Fig. 2K, see top panel). When grown in the presence of inducer, overexpression of *ypsA-gfp* resulted in a severe growth defect (Fig. 2K, see bottom panel). However, growth was similar to WT in all intragenic suppressor strains when grown in the presence of inducer (Fig. 2K, see bottom panel). Given that these mutants were unable to cause filamentation, it appears that the lethality is directly linked to the ability of YpsA to elicit filamentation. Collectively, these data indicate that the residues E55, P79, R111, and G132 are critical for the function of YpsA, especially in regard to cell division inhibition.

### Structural analysis of the intragenic suppressor mutations.

**FIG 3:**
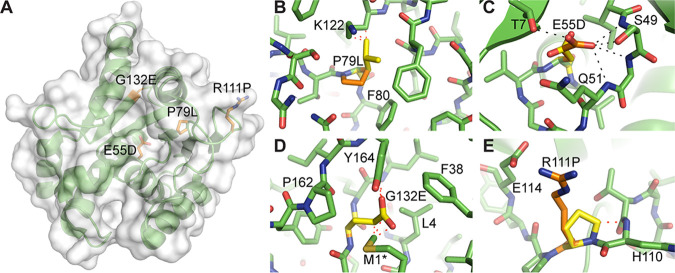
Structural analysis of the intragenic suppressors. (A) Crystal structure of B. subtilis YpsA (PDB ID 2NX2) with sites of mutation colored in orange. (B to E) Computationally generated mutants are shown in yellow. Hydrogen bonds are shown as black dashes, while steric clashes are represented as red dashes. For clarity, only the most severe clashes are indicated with interatomic distances of <2.2 Å. (B) The P79L mutation generates severe clashes with multiple surrounding residues. (C) The E55D mutation in the putative DNA-binding groove results in the potential loss of five hydrogen bonds, destabilizing this region. (D) The G132E mutation, similar to P79L mutant, involves a core residue that cannot accommodate any large side chains without severe steric clashes. The asterisk (*) designates the modeled conformation of M1, which was converted from the nonnative L1 in the original deposited structure (see Materials and Methods for a description). (E) The R111P mutant eliminates a salt bridge with E114 and produces a clash with the adjacent H110 backbone. As a surface residue, it also potentially disrupts intermolecular interactions and signaling.

Three of the four YpsA mutants have residues that are buried in the core: E55D, G132E, and P79L (Fig. 3A). When large mutations occur in this environment, misfolding and loss of function is often the consequence (12). Among these mutants, P79 is significant because it, and its adjacent residue F80, are strictly conserved among the YpsA clade of *Firmicutes* (7, 9). The crystal structure of B. subtilis YpsA (PDB ID 2NX2) reveals P79 and F80 also line the possible DNA binding groove of YpsA (8). The positioning of an aromatic side chain here suggests it may facilitate DNA binding by forming stacking interactions with nucleobases (13). The P79L mutation not only creates severe clashes with surrounding residues but likely perturbs the positioning of F80 and therefore may directly impair DNA or nucleotide binding (Fig. 3B). Likewise, the E55D mutant affects a second, highly conserved segment of the putative DNA binding domain, the GxE motif. In YpsA, E55 is highly coordinated by five potential hydrogen bonds with the side chain and backbone of S49, the T7 side chain, and the backbone amide of Q51 (Fig. 3C). Considering the size and physicochemical properties of their side chains, one would expect an E→D mutation to have a nondeleterious effect on YpsA. However, in this instance, shortening the side chain of E55 by a methylene results in the weakening or total loss of these hydrogen bonds and likely destabilizes the possible DNA binding groove. In addition, the aliphatic part of the E55 side chain forms extensive van der Waals interactions with nearby residues such as Q51 and others, and the carboxylate group of an aspartate residue at this position would also clash with these surrounding residues. The third core mutation, G132E, is located at the beginning of a β-strand and is surrounded by multiple bulky and hydrophobic residues, including Y164, P162, M1*, L4, and F38 (Fig. 3D). Conversion from glycine to any other residue besides alanine results in clashes that will affect the secondary structural elements from which the surrounding residues originate. Indeed, every possible G132E rotamer produces significant clashes, with interatomic distances of <2.2 Å.

The R111P mutation is the only intragenic suppressor mutation that involves a solvent-exposed residue. Here, R111 normally forms a salt bridge with the neighboring E114 (Fig. 3E). The conversion from a positively charged side chain to a nonpolar one eliminates this interaction. Furthermore, the cyclic nature of the proline side chain introduces a steric clash with the backbone amide nitrogen of its adjacent residue, H110. The R111P mutation will likely force conformational changes in the protein backbone and cause significant disruptions in intramolecular interactions involving nearby residues. A second scenario that leads to the disruption of YpsA function in this mutant involves the impairment of intermolecular interactions and macromolecular recognition. It is possible that a mutation from R→P prevents interaction with other protein partners of YpsA.

### Isolation and validation of an extragenic suppressor mutation in *yfhS*.

Using the same suppressor screening approach (Fig. S2), we were able to isolate and validate an extragenic suppressor mutation, which is a duplication of a stretch of 10 nucleotides that introduces a premature stop codon in *yfhS* (Fig. S3A). YfhS is a 74-amino-acid protein of unknown function. *yfhS* is annotated as a sporulation gene upregulated by SigE sigma factor during sporulation (14). However, there is no sporulation defect in a *yfhS*-null strain (15). *yfhS* may also be regulated by the transcription factor AbbA (16).

10.1128/mSphere.00655-20.3 FIG S3 Analysis of *yfhS* extragenic suppressor mutation. (A) Pairwise alignment of the *yfhS* sequence in WT (PY79) and the extragenic suppressor (RBSS6E11). The source of 10-nucleotide duplication is highlighted. (B) Growth curves of WT (PY79), Δ*yfhS* (RB314), Δ*yfhS* + *yfhS* (RB409; grown in 250 μM IPTG), Δ*ypsA* Δ*yfhS* (RB420), and Δ*ypsA* Δ*yfhS* + *ypsA* (RB433; grown in 250 μM IPTG) are shown. (C) Spot titer assay of WT (PY79), Δ*ypsA* (RB42), Δ*yfhS* (RB314), Δ*ypsA* Δ*yfhS* (RB420), or strains containing an inducible copy of *ypsA* in either a WT background (GG82; YpsA) or in a Δ*ypsA* Δ*yfhS* background (RB433). Dilutions of standardized cultures were spotted on solid medium without inducer (left panel) or containing 1 mM IPTG (right panel). Download FIG S3, PDF file, 2.0 MB. Copyright © 2020 Brzozowski et al. 2020 Brzozowski et al. This content is distributed under the terms of the Creative Commons Attribution 4.0 International license.

**FIG 4:**
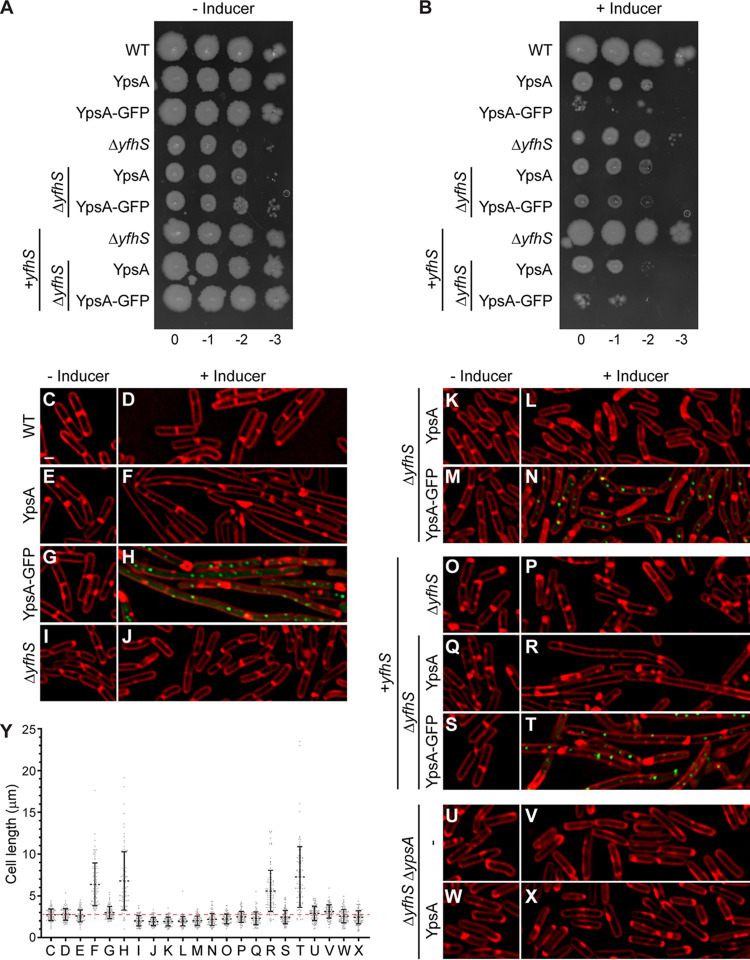
Deletion of *yfhS* rescues YpsA-mediated toxicity and associated filamentation. (A and B) Spot assay of WT cells (PY79), Δ*yfhS* cells (RB314), Δ*yfhS* + *yfhS* cells (RB409), and cells overexpressing either *ypsA* or *ypsA-gfp* in an otherwise wild-type background (GG82 and GG83), a Δ*yfhS* background (RB288 and RB289), or in a Δ*yfhS* complementation strain where an intact copy of *yfhS* is engineered to be under the control of an IPTG-inducible promoter at an ectopic locus (RB410 and RB411). Cultures were standardized, and serial dilutions were spotted onto solid medium without inducer (A) or with 1 mM IPTG (B). Corresponding dilution factors are indicated on top. (C to X) Fluorescence microscopy comparing cell morphologies of WT cells (PY79), Δ*yfhS* cells (RB314), Δ*yfhS* + *yfhS* cells (RB409), and cells overexpressing either *ypsA* or *ypsA-gfp* in an otherwise wild-type background (GG82 and GG83), Δ*yfhS* background (RB288 and RB289), Δ*yfhS* complementation strain (RB410 and RB411), or Δ*ypsA* Δ*yfhS* background (RB420 and RB433). Cells were imaged in the absence of inducer (C, E, G, I, K, M, O, Q, S, U, and W) or in the presence of 250 μM IPTG for 1 h (D, F, H, J, L, N, P, R, T, V, and X). The fluorescence signals of FM4-64 membrane dye (red) and GFP (green) are shown. Scale bar, 1 μm. (Y) Cell lengths of strains shown in panels C to X were quantified. The corresponding mean values and standard deviations (*n* = 100) are shown. A red dotted line serves as a reference to the average WT cell length.

To test whether disruption of *yfhS* restores normal cell length in cells overexpressing either *ypsA* or *ypsA-gfp*, we generated a strain harboring an inducible copy of either *ypsA* or *ypsA-gfp* in a *yfhS*-null background. These strains were then screened with the appropriate controls via a spot assay in order to observe whether or not the *yfhS* deletion was able to restore normal growth on solid medium even when YpsA or YpsA-GFP was overproduced. In the absence of inducer, WT cells and cells containing an inducible copy of either *ypsA* or *ypsA-gfp* grew similarly (Fig. 4A). Cells lacking *yfhS* formed small colonies in comparison to WT, suggesting an intrinsic growth phenotype associated with the deletion of *yfhS*. Cells harboring an inducible copy of *ypsA* or *ypsA-gfp* in a *yfhS*-null background grew similarly to the *yfhS*-null control strain. When grown in the presence of inducer, as shown in Fig. 1B, cells harboring an inducible copy of *ypsA* showed a moderate growth defect while inducible *ypsA-gfp* strain exhibited a severe growth defect (Fig. 4B). In the presence of inducer, cells harboring a *yfhS* knockout and cells harboring a *yfhS* knockout with an inducible copy of either *ypsA* or *ypsA-gfp* grew similarly, suggesting that deletion of *yfhS* prevents elicitation of lethal phenotypes displayed by YpsA or YpsA-GFP overproducing cells (Fig. 4B). To ensure the phenotype was specific to the disruption of the native copy of *yfhS*, we introduced *yfhS* at an ectopic locus under an inducible promoter. In this complementation strain, the presence of inducer or even leaky expression in the absence of inducer restored WT-like growth (Fig. 4A). The defective growth phenotype of YpsA- and YpsA-GFP-overproducing cells was also restored in the presence of inducer in the complementation strain (compare Fig. 4A and B).

Interestingly, upon deletion of both *ypsA* and *yfhS*, the Δ*yfhS* plate phenotype resembled that of WT and Δ*ypsA* (Fig. S3C), suggesting that the *yfhS*-null phenotype is dependent on the presence of YpsA. To test whether the disruption of YpsA-mediated toxicity in *yfhS*-null strain is linked to the slow growth phenotype or YfhS, we engineered *ypsA* to be expressed under the control of an inducible promoter from an ectopic locus in a Δ*ypsA* Δ*yfhS* strain background. In the presence of inducer, we observed normal growth even upon *ypsA* overexpression (Fig. S3C; compare YpsA and Δ*ypsA* Δ*yfhS* plus YpsA strains). This indicates that YpsA-mediated lethality requires YfhS and that the lethal phenotype is not due to slow growth of *yfhS* (Fig. 4B), since we also observed the reversal of lethality in Δ*ypsA* Δ*yfhS* strain that displays normal growth.

Next, we inspected the cell morphology of all strains tested in Fig. 4A and B through fluorescence microscopy. Cell division in cells harboring an inducible copy of either *ypsA* or *ypsA-gfp*, but not in the WT control, were inhibited upon the addition of inducer (Fig. 4C to H), as discussed previously (Fig. 1A) (7). The quantification of cell lengths is shown in Fig. 4Y. The cell lengths of the WT control strain in the absence and presence of inducer were similar (WT [–inducer], 2.72 ± 0.68 μm [Fig. 4C]; WT [+inducer], 2.77 ± 0.69 μm [Fig. 4D]). In contrast, as expected, cells overproducing YpsA or YpsA-GFP exhibited filamentation in the presence of inducer (YpsA [–inducer], 2.59 ± 0.71 μm (Fig. 4E); YpsA [+inducer], 6.36 ± 2.56 μm [Fig. 4F]; YpsA-GFP [–inducer], 2.99 ± 0.73 μm [Fig. 4G]; YpsA-GFP [+inducer], 6.77 ± 3.48 μm [Fig. 4H]).

Upon imaging the Δ*yfhS* cells, we noticed that the average cell length was smaller than for WT cells (Δ*yfhS* [–inducer], 1.99 ± 0.61 μm; compare Fig. 4I and C). In addition to a smaller cell length, the average cell width of Δ*yfhS* cells also appeared to be smaller compared to WT (WT [–inducer], 0.78 ± 0.06 μm [Fig. 4C]; Δ*yfhS* [–inducer], 0.67 ± 0.08 μm [Fig. 4I]; *n* = 100). This observation hints at the possible role for YfhS in cell size regulation either directly or indirectly. The addition of inducer had no effect on the average cell length of cells lacking *yfhS* (Δ*yfhS* [+inducer], 1.91 ± 0.48 μm; Fig. 4J). When cells containing an inducible copy of either *ypsA* or *ypsA-gfp* in a *yfhS*-null background were imaged, they also exhibited smaller cell lengths in the absence of inducer (Δ*yfhS* + YpsA [–inducer], 1.93 ± 0.52 μm [Fig. 4K]; Δ*yfhS* + YpsA-GFP [–inducer], 2.02 ± 0.55 μm [Fig. 4M]), suggesting that that small-cell phenotype is intrinsically linked to the lack of the *yfhS* gene. Intriguingly, overproduction of either YpsA or YpsA-GFP did not result in filamentation in a *yfhS*-null background (Δ*yfhS* + YpsA [+inducer], 1.97 ± 0.56 μm [Fig. 4L]; Δ*yfhS* + YpsA-GFP [+inducer], 2.17 ± 0.72 μm [Fig. 4N]), indicating that YpsA-mediated cell division inhibition is dependent on YfhS. The ratios of YpsA-GFP-FLAG and SigA in the WT background and the *yfhS*-null background were similar (0.68 and 0.56, respectively; Fig. S1D), suggesting that the elimination of cell division inhibition is not due to defective accumulation of YpsA-GFP. We did observe a 2-fold increase in the ratio of YpsA-FLAG and SigA between the WT and Δ*yfhS* backgrounds (0.80 and 1.59, respectively; Fig. S1D). However, this can be attributed to lower levels of SigA that we have seen reproducibly in this strain background, when the optical density (OD) is standardized between the strains tested. Thus, we conclude that the abolition of cell division inhibition in *yfhS*-null strain is not due to defective accumulation of YpsA or YpsA-GFP.

We further confirmed that the *yfhS* deletion phenotype is linked specifically to *yfhS* and not due to any kind of polar effect by using the complementation strain described earlier. Fluorescence microscopy revealed that the characteristic small-cell phenotype of Δ*yfhS* was no longer observed, even in the absence of inducer, likely due to the leaky expression of *yfhS* in the complementation strain (Δ*yfhS* + *yfhS* [–inducer], 2.23 ± 0.58 μm [Fig. 4O]). When the expression of ectopic *yfhS* was induced by the addition of inducer, the average cell length resembled that of the WT control (Δ*yfhS* + *yfhS* [+inducer], 2.47 ± 0.65 μm; compare Fig. 4P and D). As expected, cells carrying an IPTG-inducible copy of *ypsA* or *ypsA-gfp* in the complementation strain background, in the absence of inducer, appeared to be similar to the WT control (Δ*yfhS* + *yfhS* + YpsA [–inducer], 2.31 ± 0.76 μm [Fig. 4Q]; Δ*yfhS* + *yfhS* + YpsA-GFP [–inducer], 2.44 ± 0.80 μm [Fig. 4S]). However, in the presence of inducer, filamentation was restored in these two strains (Δ*yfhS* + *yfhS* + YpsA [+inducer], 5.60 ± 2.47 μm [Fig. 4R]; Δ*yfhS* + *yfhS* + YpsA-GFP [+inducer], 7.25 ± 3.64 μm [Fig. 4T]), confirming that YpsA-mediated filamentation requires YfhS.

Consistent with the plate phenotype, deletion of *ypsA* and *yfhS* led to the restoration of normal WT-like cell size (Δ*yfhS* Δ*ypsA* [–inducer], 2.90 ± 0.85 μm [Fig. 4U]; Δ*yfhS* Δ*ypsA* [+inducer], 3.12 ± 0.79 μm [Fig. 4V]). In this *yfhS ypsA* double-knockout background, the overexpression of *ypsA* does not lead to filamentation, suggesting that YpsA-mediated filamentation is strictly dependent on the presence of YfhS and is not related to the cell size defect seen in *yfhS*-null background. In fact, a decrease in the average cell length was observed when *ypsA* was introduced in *trans* (Δ*yfhS* Δ*ypsA* + YpsA [–inducer], 2.56 ± 0.81 μm [Fig. 4W]; Δ*yfhS* Δ*ypsA* + YpsA [+inducer], 2.43 ± 0.78 μm [Fig. 4X]), indicating that the small cell phenotype in the *yfhS*-null strain is linked to the presence of YpsA. However, the precise reason for this requirement is unclear at this time.

## DISCUSSION

Although many factors involved in facilitating the cell division process have been discovered in B. subtilis (3) and E. coli (2), our understanding is still incomplete even in these model organisms since evidence of yet-to-be-uncovered factors exists (5, 6). We reported previously that YpsA is such a factor, which appears to play a role in cell division in B. subtilis and S. aureus (7). The precise mechanism by which YpsA functions remains unclear. The structure of YpsA and another SLOG superfamily member DprA, a single-stranded DNA binding protein, is similar. Thus, it is possible YpsA also binds DNA or nucleotides such as NAD or ADP-ribose, as speculated previously (7). We undertook this study to shed light on the possible pathways through which YpsA functions. In this report, we describe our observations of YpsA-mediated lethality on solid medium and utilized that phenomenon as a tool to isolate spontaneous suppressors. Using this technique, we have isolated intragenic suppressors and an extragenic suppressor that abolishes YpsA-mediated toxicity.

We have isolated four intragenic suppressors (E55D, P79L, R111P, and G132E) using our screen. Given that E55 and P79 residues are highly conserved among YpsA, perhaps not surprisingly, mutations in those residues render YpsA inactive at least with respect to its potential function in cell division. It appears that in the E55D mutation, even though it retains the negative charge, shortening of the side chain appears to weaken the ability to form hydrogen bonds with neighboring residues. The mutations in highly conserved P79 and weakly conserved G132 residues create several steric clashes, which explains why the function of YpsA in cell division is affected. The mutation in the solvent-exposed residue R111 may result in weakened intramolecular and/or intermolecular interactions. Thus, our screen has identified multiple key residues that are essential for YpsA-mediated filamentation in B. subtilis. It is important to note that E55D mutation allows YpsA-GFP focus formation (Fig. 2J), while the negative charge reversal in an E55Q mutation did not (7). Both E55D and E55Q mutants are unable to elicit filamentation upon overproduction. Given that the E55 residue belongs to the predicted substrate binding pocket of YpsA (9), we are able to speculate that the negative charge at the binding pocket is important for substrate binding.

The extragenic suppressor mutation we isolated introduced a premature stop codon in the *yfhS* open reading frame. YfhS is a relatively small protein (74 amino acids) of unknown function. *yfhS* is upregulated during sporulation through the SigE transcription factor (15) and possibly by AbbA (16); thus, it has been classified as a sporulation gene. In our results, we note that Δ*yfhS* cells appear smaller in both width and length compared to our WT control, suggesting that YfhS may have a role in cell size regulation during vegetative growth. Furthermore, we tested and confirmed that YpsA-mediated cell division inhibition is dependent on the presence of full-length YfhS.

**FIG 5:**
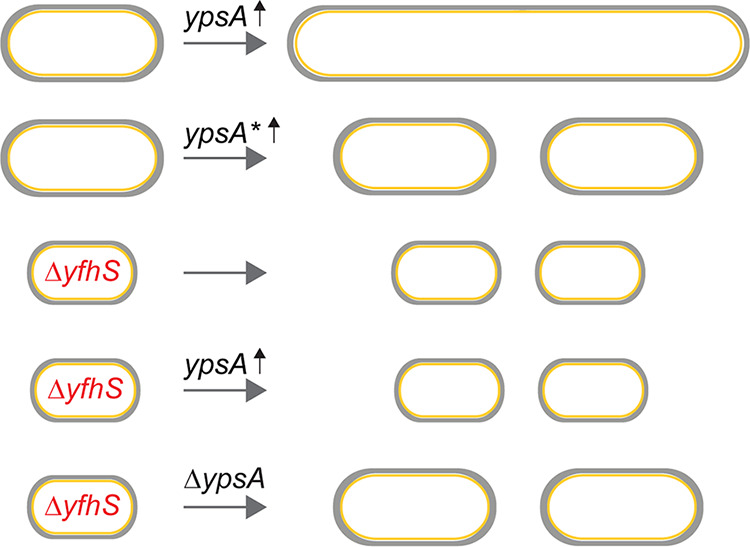
Summary of our key findings. Increased expression of *ypsA* (*ypsA* ↑) results in cell division inhibition (filamentation) and impairment of colony formation on solid medium. Colonies that do arise possess compensatory suppressor mutations (ypsA*). Analysis of one such suppressor mutation led us to *yfhS*, a gene that codes for protein of unknown function. YfhS appears to a play a role in regulating cell length and cell width. Deletion of *yfhS* abrogates the YpsA-mediated cell division phenotype, and deletion of *ypsA* disrupts the YfhS-associated cell size phenotype.

Given that YfhS is a protein of unknown function, how YfhS and YpsA are linked remains to be determined. During our course of experiments, we noticed that Δ*yfhS* cells grew slower than our WT control (Fig. S3B). We have previously shown that YpsA-mediated cell division inhibition is a growth-rate-dependent phenomenon (7); thus, we wondered whether the abolition of cell division inhibition in cells lacking *yfhS* could also be attributed to slow growth. However, given that the disruption of filamentation is also noted in *ypsA yfhS* double-knockout strain, which shows normal WT-like growth pattern (Fig. S3B), we think the dependency of YpsA-mediated filamentation on *yfhS* is likely not due to the low growth rate seen in Δ*yfhS* strain. Also, based on the fact that Δ*yfhS* cell size and plate phenotypes could be reversed by the deletion of *ypsA* (Fig. S3B; see also Fig. 4C, I, J, U, and V), we cannot rule out a direct mechanistic link between YpsA and YfhS. The key findings in this report are summarized in Fig. 5.

## MATERIALS AND METHODS

### Strain construction and general methods.

All B. subtilis strains utilized during the course of this study are derivatives of the laboratory strain PY79 (17). Table S1 contains all relevant strain and oligonucleotide information. The construction of strains overexpressing *ypsA*, *ypsA-gfp*, *ypsA-flag*, and *ypsA-gfp-flag* has been described previously (7). In order to construct a B. subtilis strain containing an inducible copy of *yfhS*, *yfhS* was PCR amplified from PY79 chromosomal DNA using the primer pair oRB59/oRB60. The resulting PCR product was digested with SalI and NheI restriction enzymes and cloned into pDR111 (D. Rudner), also digested with SalI and NheI, to construct plasmid pRB54. The constructed plasmids were then transformed into competent PY79 cells to introduce genes of interest via double-crossover homologous recombination into either the native and nonessential *amyE* locus or into a second *amyE* locus (*bkdB*::Tn*917*::*amyE*::*cat*; Amy Camp).

10.1128/mSphere.00655-20.4 TABLE S1 Strains and oligonucleotides used in this study. Download Table S1, PDF file, 0.1 MB. Copyright © 2020 Brzozowski et al. 2020 Brzozowski et al. This content is distributed under the terms of the Creative Commons Attribution 4.0 International license.

### Media and culture conditions.

Overnight B. subtilis cultures were grown at 22°C in Luria-Bertani (LB) growth medium and subsequently diluted 1:10 into fresh LB medium. Cultures were grown at 37°C in a shaking incubator to mid-logarithmic-growth phase (OD_600_ = 0.5), unless otherwise stated. In order to induce the expression of genes under the control of an IPTG-inducible promoter, 250 μM IPTG was added to growing cultures, where required, at mid-logarithmic phase, unless stated otherwise.

### Spot assay.

All spot assays were completed on LB agar plates supplemented with 1 mM IPTG, where required, to induce the expression of genes under the control of an IPTG-inducible promoter. Required strains were first grown to mid-logarithmic phase (OD_600_ = 0.5) at 37°C while shaking and subsequently standardized to an OD_600_ of 0.1. After standardization, serial dilutions of each of the strains were spotted onto the appropriate LB plates at a volume of 1 μl. Plates were incubated overnight (∼14 h) at 37°C. On the following day, the plates were observed for growth defects.

### Isolation of spontaneous suppressor mutations.

The severe growth defect associated with the strain overproducing YpsA-GFP (GG83) allowed for the isolation of spontaneous suppressor mutations that were able to restore growth similar to the WT control. Suppressor mutations were isolated and determined to be either intragenic or extragenic, as indicated in Fig. S2. For this purpose, the strain GG83 was plated on LB agar plates containing 1 mM IPTG to induce the expression of *ypsA*-*gfp*, and plates were incubated overnight at 37°C. After overnight incubation, the plates were examined for growth defects associated with the overproduction of YpsA-GFP. PY79 was utilized as a control to ensure that any reduction in growth was specifically due to the overproduction of YpsA-GFP. Single colonies of GG83 that did arise in the presence of inducer (likely containing suppressor mutations) were isolated from the original plate and used to inoculate new LB agar plates that were then grown overnight at 37°C. Genomic DNA was then isolated from each of the strains containing suppressor mutations using standard phenol-chloroform DNA extractions. Isolated genomic DNA was then used to transform WT PY79 cells, which were then screened for integration of *ypsA-gfp* into the nonessential *amyE* locus. The resulting transformants were then inoculated onto LB agar plates supplemented with 1 mM IPTG to induce the expression of *ypsA-gfp*, and the plates were incubated overnight at 37°C. On the following day, the plates were screened for growth defects associated with *ypsA-gfp* overexpression. PY79 was used as a control to ensure that any observed growth defect was specifically due to the production of YpsA-GFP. If strains harboring an inducible copy of *ypsA-gfp* isolated during our suppressor screen were now able to grow in the presence of the IPTG inducer, then the suppressor mutations were noted as possibly intragenic (ypsA*-gfp). If the strains were still unable to grow in the presence of the IPTG inducer, then the mutations were labeled as possibly extragenic, since this indicated that the inducible copy of *ypsA-gfp* within the *amyE* locus did not contain any mutations that were able to restore WT-like growth. All strains determined to contain intragenic suppressor mutations within the IPTG-inducible copy of *ypsA-gfp* at the *amyE* locus were screened via fluorescence microscopy to ensure GFP fluorescence, ruling out some potential mutations within the promoter region, frameshift mutations, and introduction of premature stop codons. Genomic DNA was isolated from each of the ypsA*-gfp strains containing intragenic suppressor mutations and was then used as a template for PCR using primer pair oP106/oP24 to amplify the *ypsA-gfp* within the *amyE* locus. The resulting PCR products were sequenced using a 3′ internal GFP sequencing primer (oP212) by Genewiz (South Plainfield, NJ). Sequence analysis was completed using ApE Plasmid Editor (v2.0.51; M. Wayne Davis), and multiple sequence alignments were built using Clustal Omega multiple sequence alignment software (18). All strains characterized as containing extragenic suppressor mutations that restored WT-like growth to strains overproducing YpsA-GFP were subjected to additional screening prior to whole-genome sequencing by integrating a new copy of *ypsA-gfp* into the *amyE* locus. The original copy of *ypsA-gfp* was first replaced by a chloramphenicol resistance cassette, and the resulting strain was then transformed with pGG28 (7) to reintroduce a new copy of *ypsA-gfp* into the *amyE* locus. The resulting strains were then used to inoculate LB agar plates supplemented with 1 mM IPTG to verify that they were still able to grow in the presence of inducer, unlike the GG83 parental strain. PY79 and GG83 were used as controls on these plates. Plates were incubated overnight at 37°C and observed for any growth defects associated with YpsA-GFP overproduction on the following day. Genomic DNA was isolated from strains containing extragenic suppressor mutations using the Wizard Genomic DNA purification kit (Promega) and sent for whole-genome sequencing (Tufts University School of Medicine Genomics Core).

### Bioinformatics and variant detection.

Data were analyzed using CLC Genomics Workbench 11 (Qiagen Bioinformatics). First, raw reads were aligned to the PY79 reference sequence (CP00681) using the Map Reads to Reference tool. Output read mappings were then subject to coverage analysis and variant detection. The Basic Variant Detection tool was used to generate a variant track and variant table output in consideration with coverage results. Resultant amino acid changes of variants unique to extragenic suppressors were examined using the Amino Acid Changes tool (v2.4) using set for genetic code parameter 11: bacterial, archaeal, and plant plasmid. Subsequently, suppressor mutations were also verified manually.

### Structural analysis.

All figures and rotamers were generated using PyMOL (Schrödinger, LLC). In the published structure (PDB ID 2NX2), the beginning residue L1 is not the native residue M1. To improve the validity of our model, we mutated L1 to M1. Briefly, the L1→M1 model was generated with the energy minimization function of UCSF Chimera using 100 descent/gradient steps and a 0.02-Å descent/step size. Charges for standard residues were generated with the AMBER ff114SB force field and the AM1-BCC force field for nonstandard residues (19–22).

### Growth curves.

PY79, RB314, and RB409 were first grown to mid-logarithmic phase (OD_600_ = 0.5) in LB broth at 37°C with shaking and subsequently standardized to an OD_600_ of 0.1. IPTG was added to the growth medium at a final concentration of 1 mM, where required, to induce the expression of genes of interest. Cultures were then grown in LB medium at 37°C with shaking for a total elapsed time of 6 h. Growth curves were plotted using Prism v8.3.1 (GraphPad Software, La Jolla, CA).

### Microscopy.

Microscopy was completed by taking 1-ml aliquots of B. subtilis cultures and washing with 1× phosphate-buffered saline (PBS) through centrifugation. Cells were then resuspended in 100 μl of PBS, and the red membrane stain FM4-64 was added at a final concentration of 1 μg/ml. The sample was prepared for microscopy by spotting 5 μl of the cell suspension onto the glass coverslip of a MatTek glass bottom dish and then covering it with a 1% agarose pad made with sterile water, as described previously (23). All imaging was completed at room temperature inside an environmental chamber using a GE Applied Precision DeltaVision Elite deconvolution fluorescence microscope. Photos were taken using a Photometrics CoolSnap HQ2 camera. All images were acquired by taking 17 z-stacks at 200-nm intervals. Images were deconvolved though the SoftWorx imaging software provided by the microscope manufacturer.

### Immunoblot analysis.

B. subtilis strains were grown overnight at 22°C in LB growth medium and then diluted 1:10 into fresh LB medium the following day. Cultures were grown to an OD_600_ of 0.5 and subsequently induced with 1 mM IPTG, where required, to induce the expression of the genes of interest. Cultures were then grown to an OD_600_ of 1.0 and, after the induction period, 1-ml aliquots of cultures were centrifuged, and cell lysis was completed by resuspending the cell pellet in a protoplast buffer containing 0.5 M sucrose, 20 mM MgCl_2_, 10 mM KH_2_PO_4_, and 0.1 mg/ml lysozyme. Samples were incubated at 37°C for 30 min and then prepared for SDS-PAGE. After electrophoresis, the samples were transferred onto a nitrocellulose membrane and subsequently probed with antibodies against GFP, FLAG (Proteintech Group, Inc.), or B. subtilis SigA (M. Fujita), which was used as an internal loading control.
