# Supplementary figures and images for: Interdependent YpsA- and YfhS-Mediated Cell Division and Cell Size Phenotypes in Bacillus subtilis
Source: mSphere. 2020 Jul 22;5(4):e00655-20. doi: 10.1128/mSphere.00655-20 (PMC7376506; doi:10.1128/mSphere.00655-20)

**A**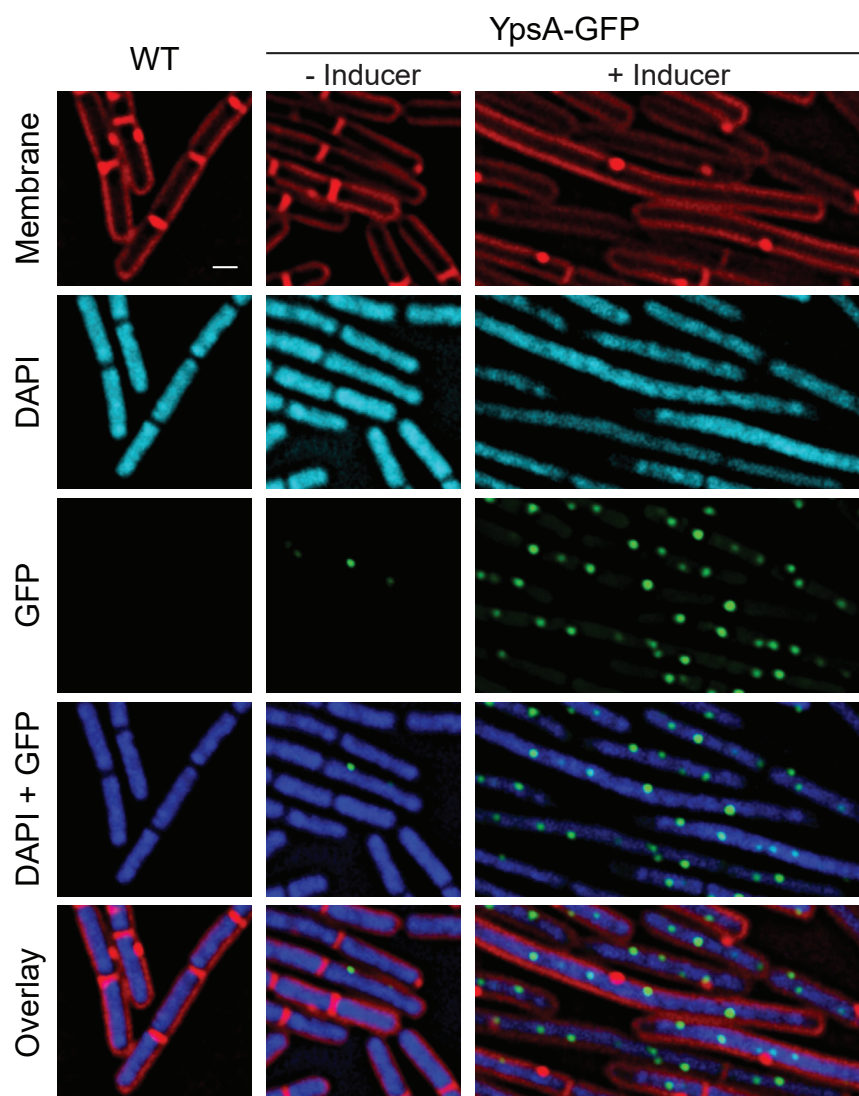**B**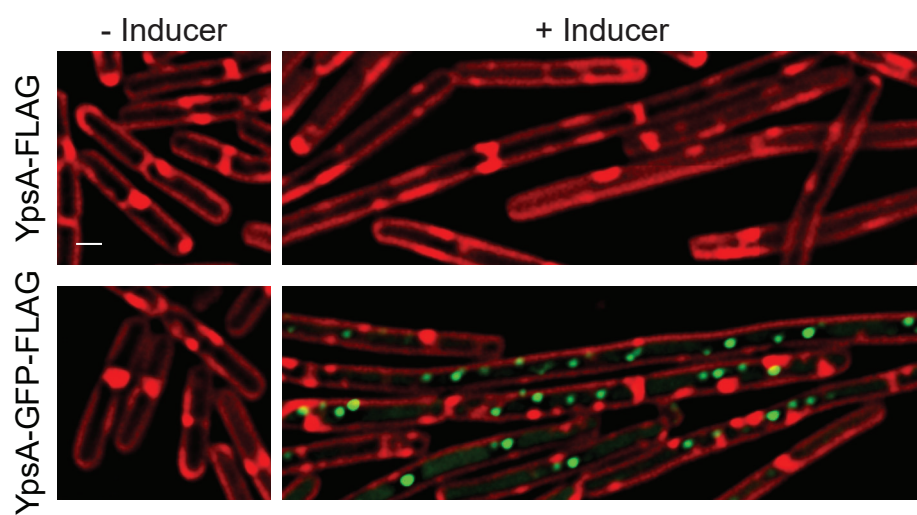**C**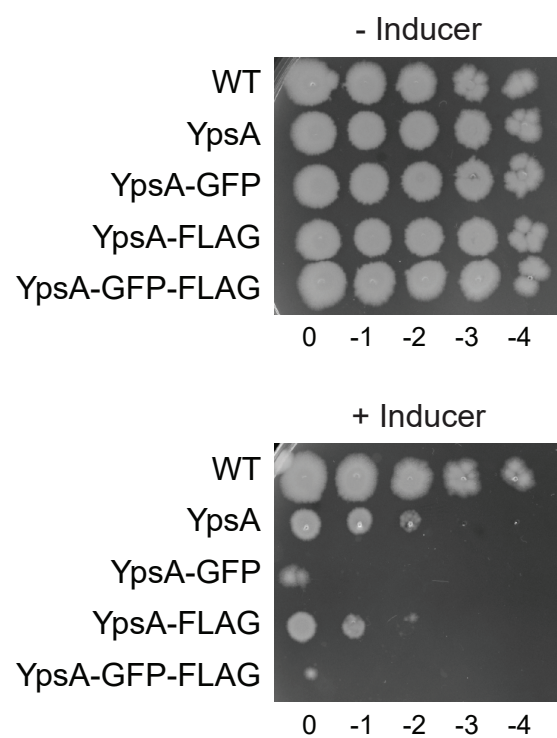**D**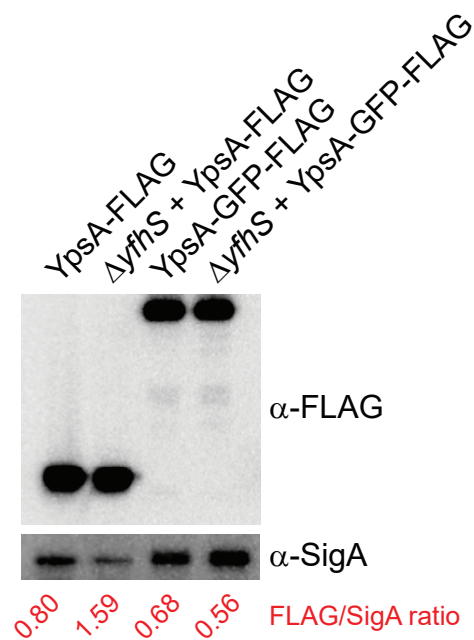

Supplement: FIG S1 [file mSphere.00655-20-sf001.pdf]

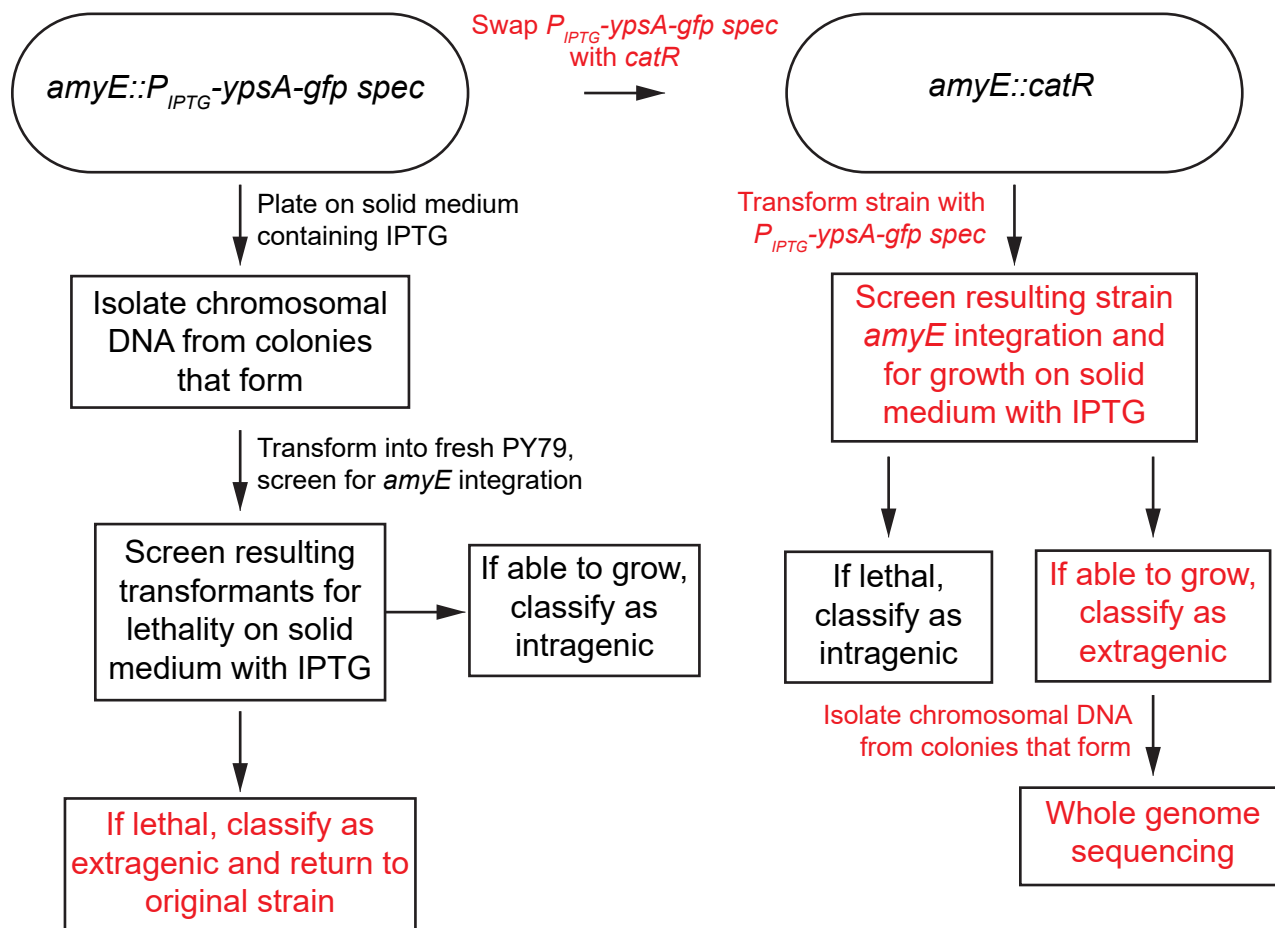

Figure S2

Supplement: FIG S2 [file mSphere.00655-20-sf002.pdf]
