# Supplementary material for: Interdependent YpsA- and YfhS-Mediated Cell Division and Cell Size Phenotypes in Bacillus subtilis
Source: mSphere. 2020 Jul 22;5(4):e00655-20. doi: 10.1128/mSphere.00655-20 (PMC7376506; doi:10.1128/mSphere.00655-20)
Supplement: FIG S3 [file mSphere.00655-20-sf003.pdf]

A

```
WT           ATGTATGTCGGACGTGATATGAGCGAATTGAACATGGTTTCCAAAAAAGATTGGAAGAAC   60
RBSS6E11     ATGTATGTCGGACGTGATATGAGCGAATTGAACATGGTTTCCAAAAAAGATTGGAAGAAC   60
             ************************************************************

WT           AGTGAACTCGCTTATTTTCATCATGCCTTTCAGCAAATTATGCCTTATTTGAACGAAGAA   120
RBSS6E11     AGTGAACTCGCTTATTTTCATCATGCCTTTCAGCAAATTATGCCTTATTTGAACGAAGAA   120
             ************************************************************

WT           GGCCAATCAAAATACCGGGAATTAACGCAAGAAATTGAAGCGCGCGGCGGAATGAAGCGC   180
RBSS6E11     GGCCAATCAAAATACCGGGAATTAACGCAAGAAATTGAAGCGCGCGGCGGAATGAAGCGC   180
             ************************************************************

WT           AATGAAG----------CGGACTACAGCCACGGCACGCGCGTCTCTTACGATTAA        225
RBSS6E11     AATGAAGCGCAATGAAGCGGACTACAGCCACGGCACGCGCGTCTCTTACGATTAA        235
             *******          **************************************
```

B

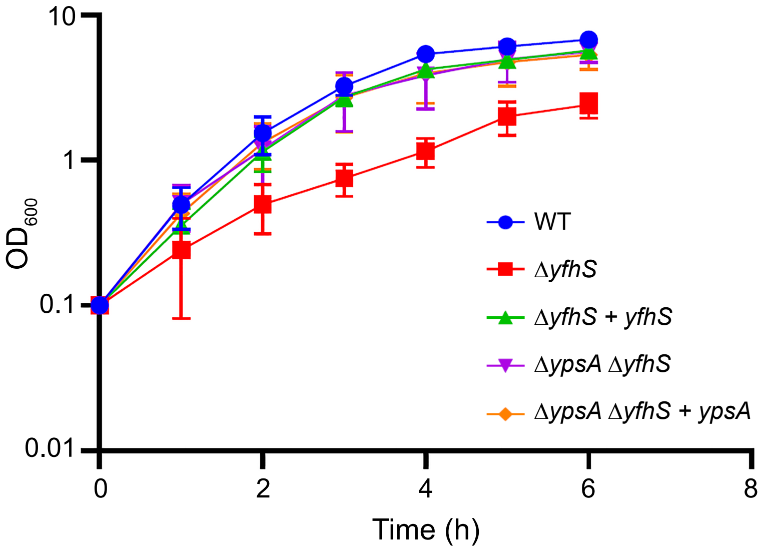

C

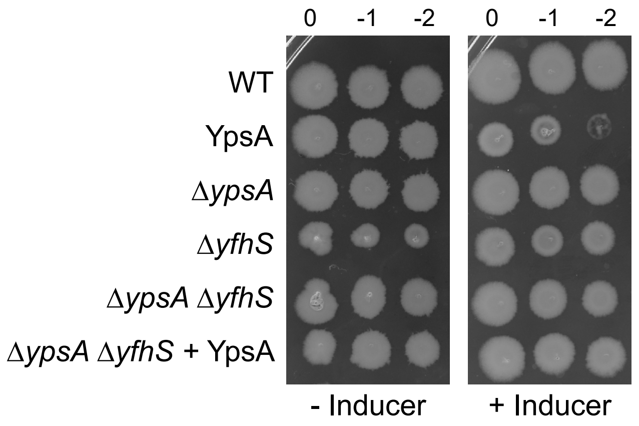

Figure S3
