# Supplementary material for: Interdependent YpsA- and YfhS-Mediated Cell Division and Cell Size Phenotypes in Bacillus subtilis
Source: mSphere. 2020 Jul 22;5(4):e00655-20. doi: 10.1128/mSphere.00655-20 (PMC7376506; doi:10.1128/mSphere.00655-20)
Supplement: TABLE S1 [file mSphere.00655-20-st001.pdf]

Table S1 Strains and oligonucleotides used in this study

Strains used in this study

Strain	Genotype	Reference
PY79	Wild type	Youngman *et al.* (1984)
GG82	*amyE::P$_{hyperspank}$-ypsA spec*	Brzozowski *et al.* (2019)
GG83	*amyE::P$_{hyperspank}$-ypsA-gfp spec*	Brzozowski *et al*. (2019)
RB121	*amyE::P$_{hyperspank}$-ypsA-flag spec*	Brzozowski *et al*. (2019)
RB125	*amyE::P$_{hyperspank}$-ypsA-gfp-flag spec*	Brzozowski *et al*. (2019)
RB300	*amyE::P$_{hyperspank}$-ypsA$_{G132E}$-gfp spec*	This study
RB301	*amyE::P$_{hyperspank}$-ypsA$_{P79L}$-gfp spec*	This study
RB327	*amyE::P$_{hyperspank}$-ypsA$_{E55D}$-gfp spec*	This study
RB328	*amyE::P$_{hyperspank}$-ypsA$_{R111P}$-gfp spec*	This study
RB314	Δ*yfhS::erm*	Derived from BKE08640 (BGSC*)
RB288	Δ*yfhS::erm amyE::P$_{hyperspank}$-ypsA spec*	This study
RB289	Δ*yfhS::erm amyE::P$_{hyperspank}$-ypsA-gfp spec*	This study
RB409	Δ*yfhS::erm bkdB::Tn917::amyE::cat::P$_{hyperspank}$-yfhS spec*	This study
RB410	Δ*yfhS::erm bkdB::Tn917::amyE::cat::P$_{hyperspank}$-yfhS spec amyE::P$_{hyperspank}$-ypsA spec::cat*	This study
RB411	Δ*yfhS::erm bkdB::Tn917::amyE::cat::P$_{hyperspank}$-yfhS spec amyE::P$_{hyperspank}$-ypsA-gfp spec::cat*	This study
RB412	Δ*yfhS::erm amyE::P$_{hyperspank}$-ypsA-flag spec*	This study
RB413	Δ*yfhS::erm amyE::P$_{hyperspank}$-ypsA-gfp-flag spec*	This study
RB420	Δ*yfhS::erm* Δ*ypsA::kan*	This study; derived from BKE08640 and BKK22190 (BGSC*)
RB433	Δ*yfhS::erm* Δ*ypsA::kan amyE::P$_{hyperspank}$-ypsA spec*	This study
RBSS6E11	*amyE::P$_{hyperspank}$-ypsA-gfp** spec*	This study

*BGSC- Bacillus Genetic Stock Center
** Strain carries suppressor mutation

Oligonucleotides used in this study

Primer	Sequence 5' to 3'
oP24	GCC<u>GCATGC</u>*TTA* TTTGTATAGTTCATCCATGCC
oP106	AAA<u>GTCGAC</u>*ACATAAGGAGGAACTACT* ATGAAAGTATTGGCAATAACGGGCTATAAACCG
oP212	GGGTAAGTTTTCCGTATGTTGCATCACCTTCACCCTCTCC
oRB59	AATAA<u>GTCGAC</u>*ACATAAGGAGGAACTACT* ATGTATGTCGGACGTGATATGAGCGAA
oRB60	AATAA<u>GCTAGC</u>*TTA* ATCGTAAGAGACGCGCGTGCCGTGGCT
